# Obituary

**Published:** 1884-12

**Authors:** 


### Obituary.

DIED.—In Indianapolis, November 4th, 1884, of pneumonia, Dr. W. M. Herriott, aged fifty-two years. Dr. Herriott's death will be a surprise to many in the profession, though he has been in rather feeble health for more than a year, yet it was not thought that he would so soon pass away. Dr. H. was widely known in the profession. He formerly, and for many years, practiced dentistry in Zanesville, Ohio, having there a large and lucrative practice which wore upon his strength so much that, ten years ago, he removed to Indianapolis, and there entered upon the dental depot business, and has in that been quite successful, being popular with all who knew him. He was a man of strict business integrity. He always manifested a great interest in his profession and in whatever would promote its prosperity. He was one of the organizers of the Ohio State Dental Society, and retained his connection with that body up to the time of his death, notwithstanding he had been absent from the State for ten years. He was always present at its meetings, and exhibited as much interest in it as those who remained in the State. He has also, since living in Indiana, been a member of the Indiana State Dental Society, and has promoted its welfare in every possible way. He was identified with the Indiana Dental College, from its organization, being a member of its Board of Trustees, and ever active in its best interests and contributing largely to its support. He was also interested in educational and benevolent work; especially was he interested in the young, with whom he was always a great favorite. He leaves a wife, two sons, and a daughter to mourn his loss. In this very great bereavement they have the warm sympathy of all who knew the Doctor. He will be missed by many of our Dental Societies, where he was usually found not as a spectator merely, but as one who took an active part in the proceedings.

